# A call for open data to develop mental health digital biomarkers

**DOI:** 10.1192/bjo.2022.28

**Published:** 2022-03-03

**Authors:** Daniel A. Adler, Fei Wang, David C. Mohr, Deborah Estrin, Cecilia Livesey, Tanzeem Choudhury

**Affiliations:** Cornell Tech, USA; Department of Population Health Sciences, Weill Cornell Medicine, New York, New York, USA; Center for Behavioral Intervention Technologies and Northwestern University Feinberg School of Medicine, Chicago, Illinois, USA; Cornell Tech, USA; Department of Psychiatry, University of Pennsylvania, Philadelphia, Pennsylvania, USA; Cornell Tech, USA

**Keywords:** Digital technology, machine learning, man–machine systems, remote consultation, mental health

## Abstract

Digital biomarkers of mental health, created using data extracted from everyday technologies including smartphones, wearable devices, social media and computer interactions, have the opportunity to revolutionise mental health diagnosis and treatment by providing near-continuous unobtrusive and remote measures of behaviours associated with mental health symptoms. Machine learning models process data traces from these technologies to identify digital biomarkers. In this editorial, we caution clinicians against using digital biomarkers in practice until models are assessed for equitable predictions (‘model equity’) across demographically diverse patients at scale, behaviours over time, and data types extracted from different devices and platforms. We posit that it will be difficult for any individual clinic or large-scale study to assess and ensure model equity and alternatively call for the creation of a repository of open de-identified data for digital biomarker development.

Over the past decade, numerous studies have explored the use of data collected from ubiquitous, everyday technologies, including smartphones, wearables, social media and computer interactions (e.g. keyboard keystrokes) to remotely and continuously measure individuals’ physiology and behaviour within the fabric of their lives. Machine learning models process this data to identify digital biomarkers associated with symptoms of depression, schizophrenia and bipolar disorder.^1^ Mental health disorders commonly remain undiagnosed or misdiagnosed, potentially owing to their heterogeneity in presentation or because non-mental health clinicians (e.g. primary care physicians), often the entry point to care, are less equipped for symptom assessment. Access to unobtrusively and remotely collected biomarkers can reduce friction to integrate physiological and behavioural data critical to understanding everyday mental health into patient care.

Tseng et al provide an example of digital biomarker identification for predicting schizophrenia symptom changes.^2^ Smartphone sensing data were collected longitudinally from 61 schizophrenia patients over the course of a year, and schizophrenia symptoms were self-reported by patients every 2–3 days. Behavioural features were calculated from raw sensing data, and machine learning models were created to predict self-reported symptoms from behavioural features. Statistical techniques uncovered behavioural features with the greatest influence on model predictions; these became candidate digital biomarkers. Although this specific example stems from the literature, medical centres have established ‘digital clinics’ to explore how digital biomarkers can best inform care.^3^

Despite the promise of digital biomarkers, it is difficult to collect both the digital and symptom outcome data required for digital biomarker construction at scale and ensure that models make consistent, equitable predictions within any single study or clinic. We define machine learning model equity as equal and accurate performance (e.g. with respect to sensitivity, specificity and positive predictive value) of a model across diverse patient subgroups over time.^4^ Without assessing model equity, introducing digital biomarkers into care is tenuous. We first describe three components of population diversity most relevant to digital biomarker model equity and explain why it is challenging for any single clinic or longitudinal study to collect data capturing all of these components (Fig. 1). We then hypothesise that an open data repository created to develop mental health digital biomarkers may solve these challenges.

**Fig. 1:**
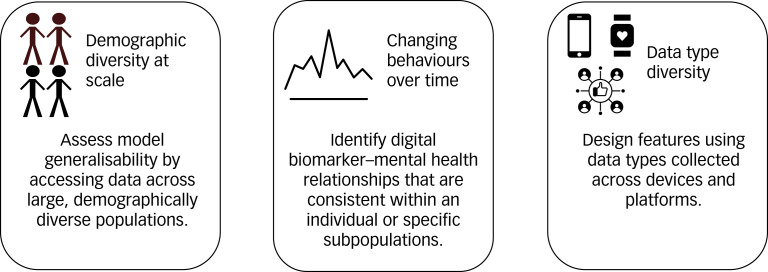
Data collection challenges that reduce the ability of any single clinic or longitudinal study to assess and ensure the model equity of digital biomarkers, and how an open data repository may address these challenges.

## Digital biomarker data collection challenges

### Demographic diversity at scale

Although previous digital biomarker research has engaged with clinical populations, it is difficult for any single clinic to recruit demographically diverse patients for digital biomarker research at scale. Clinics have easiest access to geographically collocated patients already in care and often recruit current patients into studies for digital biomarker creation.^1^ For example, Henson et al recruited 83 patients with schizophrenia in the greater Boston area to create digital biomarkers associated with psychotic relapse.^5^ Jacobson et al recruited 23 patients with a diagnosed mood disorder (major depressive disorder, bipolar I, bipolar II) in treatment to identify digital biomarkers associated with disorder diagnosis and changes in symptom severity.^6^ Recent work by Müller et al cautions against using digital biomarkers validated only within small, homogenous populations in practice: machine learning models trained using GPS-mobility features to predict depression symptoms had varying generalisation accuracy across distinct patient subgroups in a larger sample.^7^ Thus, machine learning model equity is not ensured unless digital biomarkers are validated against data collected across demographically diverse populations.

### Changing behaviours over time

Aggregate human behaviour changes over time, requiring long-term collection of digital sensor and symptom outcomes data for potential digital biomarker updates. Long-term symptom outcomes data are costly for any single clinic to collect. Digital biomarker creation requires frequent symptom sampling, typically collected through patient self-reports. Frequent self-reporting of symptoms is arduous for the patient, and thus researchers often create incentives for participation. For example, in the work by Ben-Zeev et al to develop digital biomarkers of schizophrenia, researchers collected a ten-question self-report from patients three times per week for an entire year.^8^ To encourage engagement, researchers may compensate patients per self-report collected, increasing compensation over time to retain participation.^9^ Thus, studies are careful not to overburden patients, often limiting per-patient data collection to short time periods, from 2 weeks to 3 months.^6,9^ Digital biomarker studies could rely on outcomes collected during clinical visits and recorded within medical claims databases or electronic health records (EHRs) instead of self-reports; however, medical claims and EHRs, although valuable in many contexts, provide only episodic outcome measures. Developing digital biomarkers from episodic outcomes diverges from the potential of using digital biomarkers for more frequent assessment.

Owing to limited longitudinal data collection, digital biomarkers are unlikely to generalise across time as aggregate population behaviour changes. For example, the COVID-19 pandemic isolated individuals. Studies conducted during the pandemic may uncover novel relationships among movement, social behaviour and mental health. In addition, in any year, human behaviour and mental health exhibit seasonal patterns. Machine learning models need to be trained using data that capture both seasonality and aggregate changes over time. In machine learning, this is called concept drift.^10^ Concept drift cannot be solved with large-scale studies isolated to a specific time period. Machine learning models may not be equitable for an individual over time unless behaviour remains consistent.

### Data type diversity

Individuals engage with a variety of platforms and devices. For example, smartphones and wearables are more ubiquitous in advanced economies.^11^ Data types are also inconsistent within a specific technology: Google and Apple, the companies who develop the two predominant smartphone operating systems used worldwide, have different policies on which sensor data types can be collected from smartphones hosting their operating systems. In addition, measurement errors may vary across mobile sensor hardware, and sensors evolve over time. Reviewing the cited examples, Ben-Zeev et al and Meyerhoff et al collected smartphone sensing data exclusively from Android smartphones, whereas Jacobson et al collected wristwatch actigraphy data.^6,8,9^

For digital biomarker studies leveraging social media data, platforms host many types of media (e.g. text versus photos). For example, platforms such as Instagram focus on photo and video sharing, whereas Reddit hosts primarily text-based comment threads. Thus, an individual displays different types of behaviour across different social media platforms, and may actively use only a subset of existing social media platforms. These differences manifest within platform-specific digital biomarkers. Birnbaum et al used Facebook data to identify digital biomarkers associated with relapse in schizophrenia, including an increase in co-tagging in photos and friending on the platform.^12^ Twitter data are majority text-based compared to Facebook data, and Saha et al relied on tweet keywords to identify associations with antidepressant side-effects.^13^ Equitable models need to be exposed to data types across devices and platforms, or they will only cater to subsets of the population who engage with a particular device or platform. Although a single clinic can validate digital biomarkers across multiple data types, the heterogeneity of devices and platforms internationally, many of which are country specific, make universal assessment infeasible within a clinic's local population.

## Why open data?

The model equity challenges described above can only be solved if machine learning models are trained using data collected from demographically diverse populations at scale over time, and are exposed to many different data types. Specifically, it is impractical for any single clinic or large-scale study to collect data that meaningfully capture these heterogeneities. Alternatively, we propose that researchers should publish and pool de-identified data to create digital biomarkers. This practice of publishing data-sets for shared use is called open data.

Suppose an open data repository for mental health digital biomarker construction exists. Clinicians and researchers internationally collect data from local communities and add de-identified data to the repository. This repository is updated multiple times per year from these diverse data sources, capturing behavioural changes due to seasonality. Over time, the collected data capture generational changes in behaviours and behavioural changes due to world events (e.g. the COVID-19 pandemic). New smartphones, wearables and computers are released each year, and social media platforms evolve. Researchers assess whether trained machine learning models remain accurate using data collected from newer devices and platform updates. Machine learning models trained with data from the repository are published, to be continuously evaluated and improved by the community. Clinicians serving a specific community hypothesise that current models underperform for their patients, and a paper is published validating this hypothesis. Funding is then directed to improve model performance within this population. We call to create an open data repository for mental health digital biomarkers such that this vision of equitable model development and continuous validation can be realised.

## Privacy considerations

Privacy considerations need to be addressed parallel to open data repository creation. Many interesting data types collected to create mental health digital biomarkers are revealing. For example, GPS-derived biomarkers can be an indication of both mobility and social behaviour, associated with changes in severity of depression and bipolar disorder symptoms.^1^ Collecting GPS data poses a high risk of re-identification. In addition, although individuals may have publicly facing social media profiles, users do not engage with social media with the intention of their data being collected and analysed for mental health measurement. Concerns around re-identification are imperative owing to the stigma surrounding mental illness, and legal protections for individuals who share de-identified digital biomarkers need to be upheld, given that re-identification may expose individuals involved in sensitive situations (e.g. substance misuse).

We believe that privacy is not an impediment to open data but a necessary consideration, creating interesting, unexplored research directions. First, the open data repository could be restricted to focus research on less identifiable yet clinically relevant data types, for example, activity and sleep. Second, the community could investigate using privacy-preserving techniques during data collection or model building. Machine learning techniques, including differential privacy and federated learning, attempt to limit potential re-identification while preserving the utility of the extracted data.^14^ Investing in privacy research is beneficial for protecting users who contribute to the open data repository and for the future of data-driven health research broadly.

## Building an open data repository for mental health digital biomarkers

How do we begin to build a repository? Even with de-identification, anonymisation is not guaranteed, and thus safeguards should exist to enforce ethical data usage. For example, developers of open data platforms often create transactional costs in exchange for data access. Sage Bionetworks’ Synapse is an open healthcare data-sharing platform that requires new users to undergo training, agree to and take a quiz on governance policies, and publicly disclose research motives prior to accessing data on the platform.^15^

We call on clinicians, researchers and industry experts in psychiatry, psychology, ubiquitous computing, machine learning and privacy to create a working group and decide to either use an existing platform or create a new open data platform for development of mental health digital biomarkers. In parallel, the working group should establish privacy and data standards. In the interim, the coauthors have created a webpage and provided links to two open de-identified data-sets collected by a subset of the coauthors and collaborators across Dartmouth College, the University of Washington and Northwell Health. The data-sets include smartphone sensing data and mental health outcome measures self-reported by study participants and are hosted on the Precision Behavioral Health at Cornell Tech website (https://pbh.tech.cornell.edu/data.html). We plan to update this site as more relevant data-sets become publicly available and then migrate the hosted data to the agreed-upon platform.

### Limitations

An open data repository can enable equitable digital biomarker development only if there are targeted investments to either collect or contribute data representing the different types of diversity (demographic, time, data type) described. In addition, although an open data repository will magnify existing practical challenges with respect to combining multimodal data for predictive modelling – with modalities sampled at different frequencies, carrying different statistical properties – it will also enable further research to solve these challenges, ultimately creating more robust digital biomarkers. Note that an open data repository will not resolve broader systemic biases in healthcare, which may manifest and be amplified in collected data.

## Clinical benefit and conclusion

Assessing machine learning model equity will increase clinician trust in the use of digital biomarkers for more efficient, effective and targeted care delivery, ultimately improving clinical outcomes and reducing provider burnout. Increased clinician engagement during repository creation will focus research on data types most relevant to symptom management, and sustained engagement will lead to more seamless integration of digital biomarkers into patient visits and clinical workflows.
